# Experience and technique of simultaneous robotic resection for synchronous advanced gastric and rectal cancers: a case report

**DOI:** 10.1186/s40792-020-00911-z

**Published:** 2020-07-10

**Authors:** Sachiko Kaida, Toru Miyake, Tomoharu Shimizu, Katsushi Takebayashi, Tsuyoshi Yamaguchi, Ken Ishikawa, Masaji Tani

**Affiliations:** 1 Department of Surgery, Shiga University of Medical Science, Seta Tsukinowa-cho, Otsu, Shiga 520-2192 Japan; 2 Medical Safety Section, Shiga University of Medical Science Hospital, Otsu, Shiga Japan

**Keywords:** Gastrointestinal surgery, Robotic surgery, Simultaneous resection

## Abstract

**Background:**

Recently, robotic surgery has become more common as a minimally invasive treatment for gastric cancer (GC) and rectal cancer (RC). Herein, we report successful simultaneous robotic gastrectomy and low anterior resection in a patient with advanced GC and RC.

**Case presentation:**

A 76-year-old woman who presented with bloody stool was found to have advanced GC with lymph node metastases and advanced RC. Simultaneous robotic distal gastrectomy with D2 lymph node dissection and Billroth I reconstruction and low anterior resection with D3 lymph node dissection were performed. Preoperatively, multidisciplinary medical staff discussed the case in detail and conducted a simulation with the robot, operating room, and patient. The total operative time was 648 min (console time, 520 min), and the estimated blood loss was small. The patient was discharged on postoperative day 10 without any adverse events. In this case, careful simulation of the patient cart setting and planning of the best port layout resulted in a successful surgical outcome despite this being our first simultaneous total robotic surgery for advanced GC and RC.

**Conclusions:**

Simultaneous robotic surgery for advanced GC and RC may be technically feasible and could provide an option for future minimally invasive treatment.

## Background

Recently, robotic surgery has become more common as a minimally invasive treatment for gastric cancer (GC) [1–3] and rectal cancer (RC) [4, 5], and the number of cases has been increasing. GC and colorectal cancer (CRC) are common cancers that are often simultaneously detected during gastrointestinal examination. Although there have been several reports of simultaneous excision under laparoscopic surgery, simultaneous excision of both GC and CRC by robotic surgery has rarely been reported. Herein, we report a successful simultaneous robotic distal gastrectomy and low anterior resection in a patient with advanced GC and RC. In the present study, we discuss innovations specific to robotic surgery, comparison of robotic and conventional (open and laparoscopic) methods, and their feasibility.

## Case presentation

**Fig. 1:**
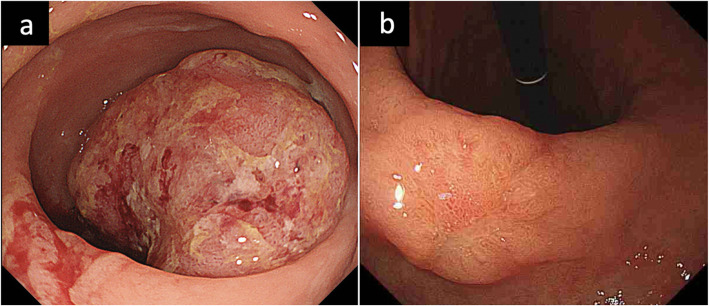
Colonoscopy and upper gastrointestinal endoscopy. **a** Colonoscopy revealed a tumor with an ulcer on the top (3 cm), located 7 cm to the oral side of the dentate line. **b** Upper gastrointestinal endoscopy revealing a 0-IIc gastric lesion on the lower gastric body

**Fig. 2:**
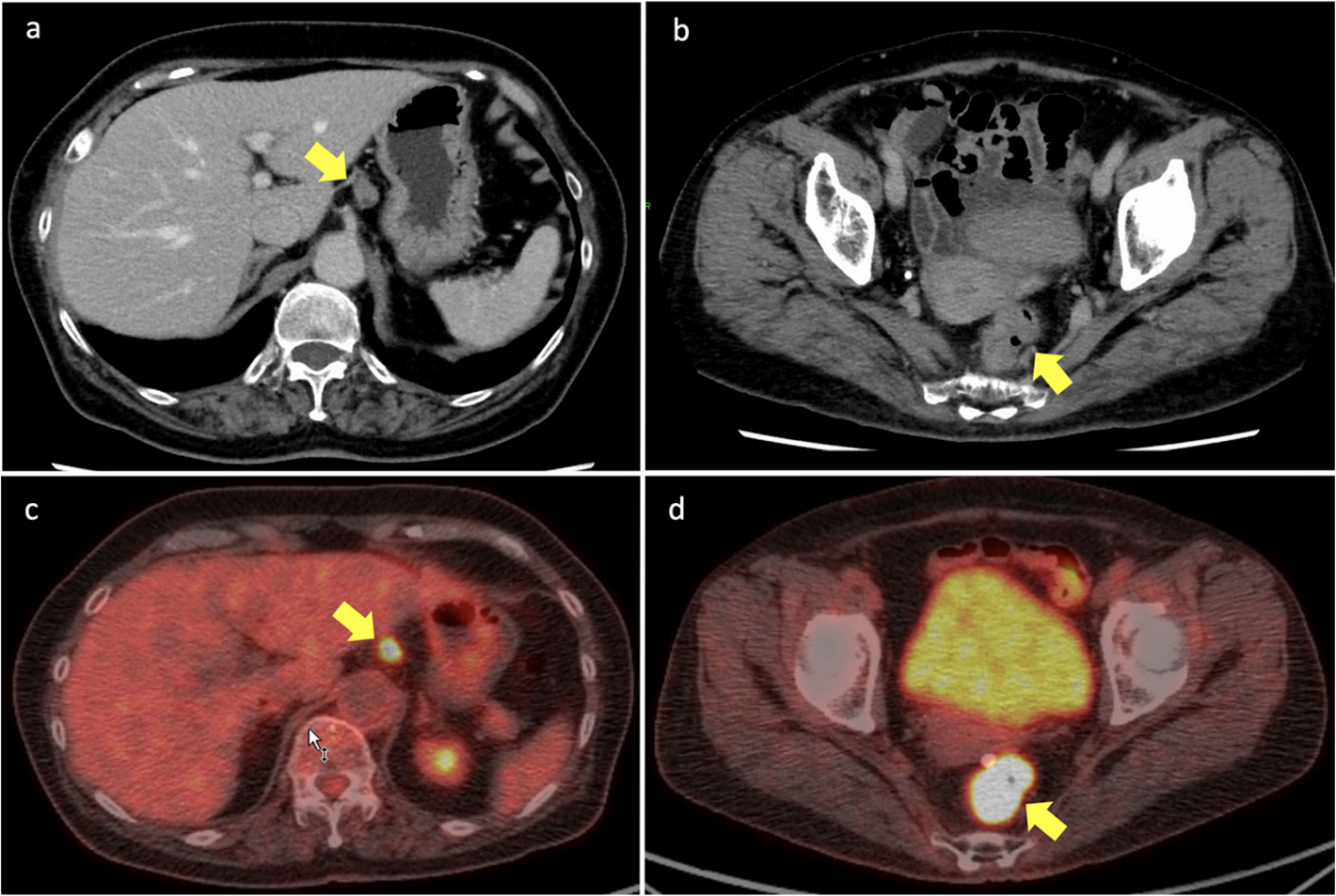
Computed tomography (CT) and fluorodeoxyglucose positron emission tomography (FDG-PET). **a** Enhanced upper abdominal CT showing three lymph node swellings in the lesser curvature area of the stomach (arrow). **b** Enhanced lower abdominal CT revealing rectal cancer invading the serosa (arrow). **c** Upper abdominal FDG-PET CT showing FDG accumulation in the lesser curvature lymph nodes of the stomach (arrow). **d** Lower abdominal FDG-PET CT scan revealing FDG accumulation in the main tumor of the rectum (arrow)

**Fig. 3:**
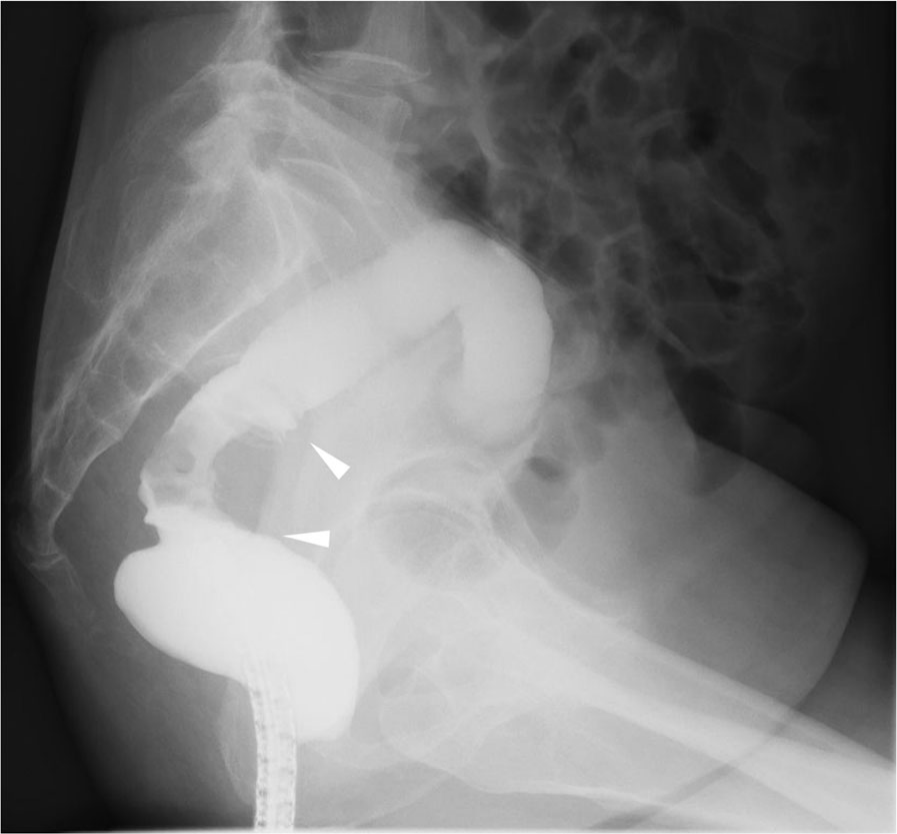
Barium contrast enema revealing the tumor site (arrow) of the rectum (Ra)

A 76-year-old woman presented with bloody stool. Colonoscopy revealed a tumor with an ulcer in the rectum, located 7 cm to the oral side of the dentate line (Fig. 1a). Endoscopic biopsy of the main rectal tumor showed pathologically well-differentiated adenocarcinoma. During upper gastrointestinal endoscopy for preoperative screening, a 0-IIc gastric lesion was revealed on the lower gastric body (Fig. 1b). Endoscopic biopsy of the gastric lesion also showed adenocarcinoma. Computed tomography (CT) showed three lymph node metastases in the lesser curvature area of the stomach (Fig. 2a) and T4a rectal cancer (Fig. 2b). Fluorodeoxyglucose (FDG) positron emission tomography CT revealed FDG accumulation in the lesser curvature lymph nodes of the stomach (Fig. 2c) and the main lesion of the rectum (Fig. 2d), but no initial distant metastasis was observed. Barium contrast enema revealed the tumor site (arrow) in the rectum (Ra) (Fig. 3). Based on these results, the patient was diagnosed with advanced GC (TNM stage T1b N2 M0: IIA) and advanced RC (Ra, TNM stage T4a N0 M0: IIB). We planned to perform simultaneous total robotic radical distal gastrectomy with D2 lymph node dissection and robotic radical low anterior resection with D3 lymph node dissection.

**Fig. 4:**
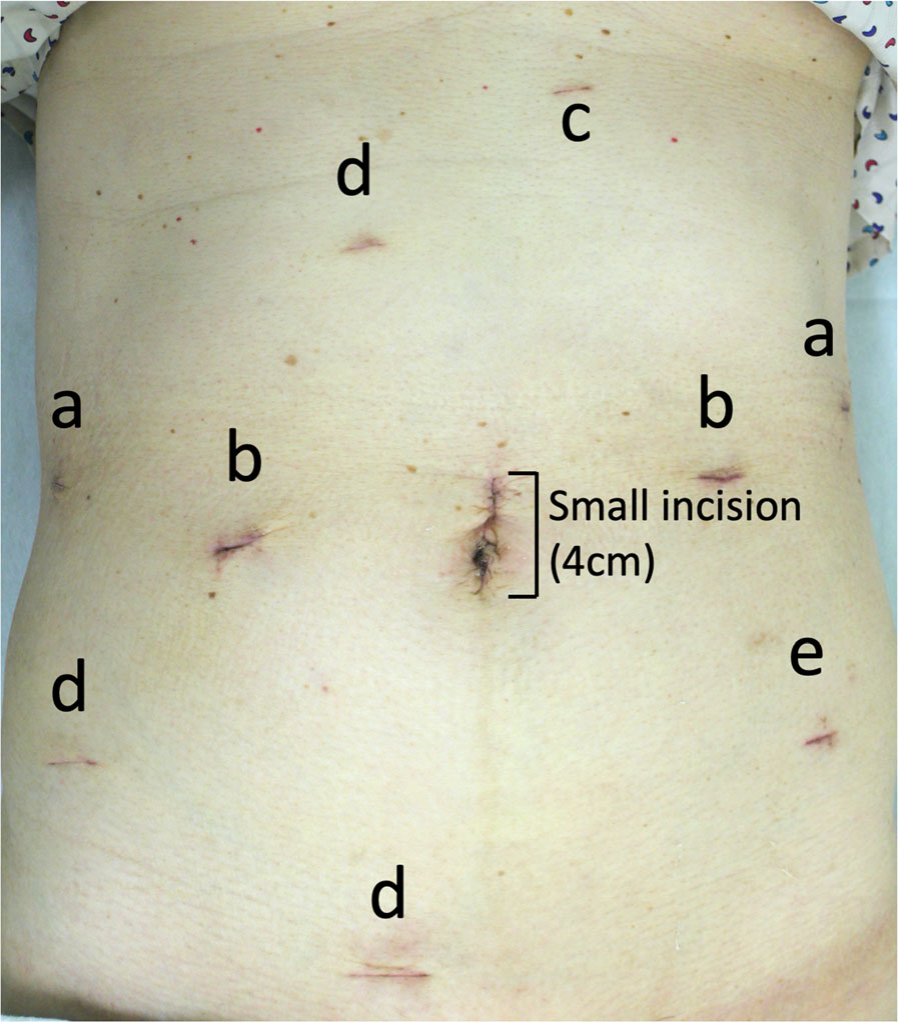
Abdominal surgical wounds on postoperative day 26. The camera port was placed using the umbilical approach. Ports (a) and (b) Port setting for robotic gastrectomy. Port (c). Wound inserted with a device for lifting the liver. Ports (b) and (d) Port setting for robotic low anterior resection. Port (e) Wound inserted with a drain for colorectal anastomosis

**Fig. 5:**
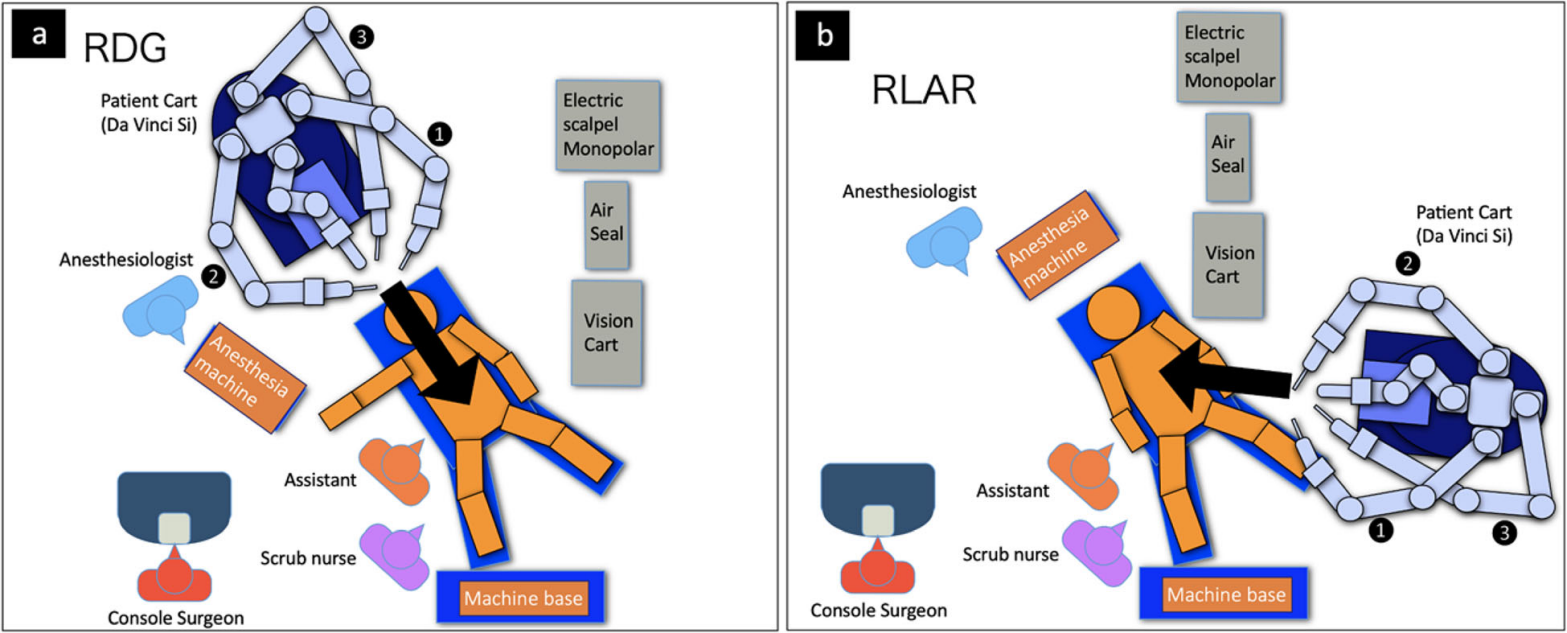
Operating room settings. **a** Operating room settings for robotic distal gastrectomy (RDG). The anesthesia machine was placed on the right side of the patient, and the patient cart was rolled from the patient’s head side. **b** Operating room settings for robotic low anterior resection (RLAR). The patient cart was moved to the caudal side through the left bedside space. The anesthesia machine was placed on the patient’s head side, and the patient cart was rolled from the left foot side of the patient. To count the number of surgeries, we only moved without turning off the power of the robot

Robotic surgery was performed by da Vinci Si (Intuitive Surgical Inc., USA), with the patient under general anesthesia and in the supine position with legs apart, for low anterior resection. The gastric and rectal cancer surgeons were different, and each of them had certifications of the console surgeons and sufficient (more than 10 cases) experiences in robotic surgery for gastric and rectal cancers. An umbilical port was inserted for the robot-scope (0°), and intra-abdominal pressure was maintained at 10 mmHg with the AirSeal® system (CONMED, Corporation, Largo, FL, USA). Robotic distal gastrectomy (RDG) with D2 lymph node dissection was initially performed using five ports (Fig. 4 ports (a) and (b)) and a Nathanson liver retractor (Fig. 4 port (c)). Preparation before robotic distal gastrectomy took 34 min (including docking of the robot [Fig. 5a]). First, the omentum was dissected in the oral direction, and the left gastroepiploic artery and vein were clipped and cut. Next, the retroperitoneum was incised at the level of the upper pancreas. The right gastroepiploic artery, vein, and gastroepiploic artery lymph nodes were dissected. Perigastric lymph nodes on the small curvature, splenic hilar lymph nodes, and pancreatic marginal lymph nodes were dissected en bloc by incising the omentum at the level of the upper pancreas and dissecting from the outer layer of the proper hepatic artery to the common hepatic artery and splenic artery. The duodenum was dissected at 1 cm on the anal side of the pylorus ring, and the stomach was dissected at the level of the marking line that was sufficiently on the oral side of the tumor using linear stapling devices (Medtronic Signia Stapling System, USA). The resected stomach was extracted through the mini-laparotomy wound (4 cm) and the extended umbilical port wound (Fig. 4). Intracorporeal gastroduodenostomy (delta-shaped anastomosis) was performed using the same linear stapling devices [6]. This part of the surgery was accomplished within 281 min (console time, 247 min), and the estimated blood loss was 0 mL.

Next, robotic low anterior resection was performed using the same two ports and three new ports (Fig. 4, ports (b) and (d)). Preparation before low anterior resection took 40 min (including undocking and docking of the robot [Fig. 5b]). Because the rectal tumor was located above the peritoneal reflection, lateral dissection was not performed during the surgical procedure [7]. Mobilization of the rectum was performed robotically. Total mesorectal excision was performed with monopolar curved scissors (da Vinci Si). Lymph nodes along the inferior mesenteric artery were dissected with high ligation. The rectum was dissected using a linear stapler (Signia™ Stapling System, Tri-staple 60 mm Purple, Medtronic, USA), and reconstruction was performed intracorporeally with a circular stapler (EEA 25 mm, Medtronic, USA). This part of the surgery was accomplished within 327 min (console time, 273 min), and the estimated blood loss was 0 mL. The transected rectum was extracted through the same umbilical incision that was used for the excision of the GC. Two drainage tubes were placed at the gastroduodenal and colorectal anastomotic sites through the port site (Fig. 4 (e)).

No complications were observed after the operation, and the patient started oral intake on the 2nd postoperative day and was discharged on the 10th postoperative day. Pathological examination revealed a moderately differentiated adenocarcinoma invading the submucosal layer with lymph node metastasis (3/35) for the GC and a well-differentiated adenocarcinoma with serosal invasion of the mucosal layer without lymph node metastasis (0/20) for the RC. A pathologically negative margin of > 3 cm was confirmed in all specimens. We assessed it as having been oncologically appropriate to perform simultaneous robotic combined resection for these cancers. Written informed consent was obtained from the patient for publication of this case report and the accompanying images.

## Discussion

We report a successful simultaneous total robotic curative resection for synchronous advanced GC and RC. Although there are some reports on simultaneous laparoscopic surgery for synchronous GC and CRC [8–11], this is the first report on simultaneous total robotic curative resection for synchronous advanced GCs and RCs. Byoung et al. first reported simultaneous robotic surgery for GC and right colon cancer [12]. Although both GC and CRC were advanced cancers in this case, robotic surgery had advantages for both peripancreatic lymph node dissection for GC surgery [13, 14] and pelvic manipulation (especially to reduce neuropathy and urinary retention) for RC surgery [4, 15] and was therefore selected to reduce postoperative complications and allow for a smooth introduction of postoperative chemotherapy for GC. In addition, simultaneous surgery may have many advantages, such as small skin incisions, reduced postoperative pain, and early mobility, resulting in decreased inflammatory cytokines and postoperative bowel obstruction [16, 17].

However, there are limitations to simultaneous robotic surgery. First, GC and RC are treated within different surgical fields, involving the upper and lower abdomen, respectively. In this case, we preoperatively examined the details of the port setting, position of the small incision, and arrangement of the operating room including the robot, anesthesia machine, nurse, and position of the patient. In a real room, we simulated the machine and the patient among surgeons, anesthesiologists, scrub nurses, and medical engineers. Additionally, the number of ports can be reduced by sharing the left and right mid-abdominal ports. We also discussed whether the gastric cancer or the rectal cancer should be resected first, and we decided to begin with the gastric cancer for the following reasons. (1) The depth of the gastric cancer was submucosal, so there was a low probability that robotic surgery would be impossible. On the other hand, the rectal cancer had the potential for extra-serosal invasion, and there was a higher probability of conversion to open LAR by incision of only the lower abdomen. (2) By performing gastric cancer surgery first, it was possible to mobilize the left side of the colon for reconstruction of the rectal cancer surgery. (3) Robotic gastrectomy and intracorporeal gastroduodenostomy (delta-shaped anastomosis) did not require mini-laparotomy. It was possible to make a small incision in favor of rectal cancer surgery. Second, the operative time was long. In previous reports, the operative time of the simultaneous robotic surgery for GC and RC was 640 min [12], and in this case, it was 648 min. Between 2013 and 2018, there were five cases of simultaneous surgery for GC and RC at our institution; two cases were open surgery, and three cases were laparoscopic surgery. The median operating time of the five cases was 631 (383–931) min, the median blood loss was 346 (0–1084) mL, and the median postoperative hospital stay was 14 (9–22) days. Among them, postoperative intestinal obstruction of grade 2 was observed in one open surgery case as a postoperative complication. Compared with these cases, the case we are comparable in postoperative short-term results. However, further analysis is required to confirm the oncological safety and feasibility of simultaneous robotic resection for synchronous advanced GCs and RCs.

We believe that simultaneous robotic surgery for synchronous advanced GC and RC may be a feasible option if these limitations can be solved by team skill and careful preoperative planning.

## Conclusions

We performed simultaneous robotic gastrectomy and robotic low anterior resection for advanced GC and RC. Simultaneous robotic surgery for gastric and rectal cancer is technically feasible and may be one of the options for future minimally invasive treatment.

## Data Availability

All data analyzed during this study are included in this article.

## Abbreviations

GC: Gastric cancer
RC: Rectal cancer
CRC: Colorectal cancer
CT: Computed tomography
FDG: Fluorodeoxyglucose
FDG-PET: Fluorodeoxyglucose positron emission tomography
RDG: Robotic distal gastrectomy
RLAR: Robotic low anterior resection
